# Neutrophils at the Crossroads: Unraveling the Multifaceted Role in the Tumor Microenvironment

**DOI:** 10.3390/ijms25052929

**Published:** 2024-03-02

**Authors:** Deepika Awasthi, Aditya Sarode

**Affiliations:** 1 Department of Obstetrics and Gynecology, Weill Cornell Medicine, New York, NY 10065, USA; 2 Department of Microbiology and Immunology, Vagelos College of Physicians and Surgeons, Columbia University, New York, NY 10032, USA

**Keywords:** neutrophils, tumor-associated neutrophils, neutrophils in cancer, metastasis, tumor microenvironment, anti-tumor neutrophils, pro-tumor neutrophils

## Abstract

Over the past decade, research has prominently established neutrophils as key contributors to the intricate landscape of tumor immune biology. As polymorphonuclear granulocytes within the innate immune system, neutrophils play a pivotal and abundant role, constituting approximately ∼70% of all peripheral leukocytes in humans and ∼10–20% in mice. This substantial presence positions them as the frontline defense against potential threats. Equipped with a diverse array of mechanisms, including reactive oxygen species (ROS) generation, degranulation, phagocytosis, and the formation of neutrophil extracellular traps (NETs), neutrophils undeniably serve as indispensable components of the innate immune system. While these innate functions enable neutrophils to interact with adaptive immune cells such as T, B, and NK cells, influencing their functions, they also engage in dynamic interactions with rapidly dividing tumor cells. Consequently, neutrophils are emerging as crucial regulators in both pro- and anti-tumor immunity. This comprehensive review delves into recent research to illuminate the multifaceted roles of neutrophils. It explores their diverse functions within the tumor microenvironment, shedding light on their heterogeneity and their impact on tumor recruitment, progression, and modulation. Additionally, the review underscores their potential anti-tumoral capabilities. Finally, it provides valuable insights into clinical therapies targeting neutrophils, presenting a promising approach to leveraging innate immunity for enhanced cancer treatment.

## 1. Introduction

Neutrophils stand out as the predominant immune cells, serving as crucial effectors in the antimicrobial response within human blood. Often referred to as polymorphonuclear cells due to their distinctive lobulated nuclei, these cells have a brief lifespan of 12–24 h [1]. The bone marrow orchestrates their constant replenishment at an astonishing rate of 10^11^ neutrophils per day, rendering them the powerhouse of the innate immune system [2]. Beyond their baseline characteristics, the lifespan of neutrophils can be altered upon activation, and the degree of activation plays a pivotal role in septic or aseptic pathology outcomes. As granulocytes, neutrophils harbor a formidable arsenal of cytotoxic factors, including antimicrobial compounds, serine proteases, lysozyme, defensins, and metalloproteases. Additionally, they possess highly intense machinery of enzymes that orchestrate oxidative bursts, endowing them with proficiency in defense mechanisms like degranulation, phagocytosis, and the release of neutrophil extracellular traps (NETs).

Advancements in techniques such as in vivo imaging, high-dimensional transcriptomic and epigenomic analyses, and studies conducted at the single-cell resolution have prompted a reevaluation of neutrophil biology in the context of cancer [3]. Notably, cancers often coincide with the recruitment of neutrophils to tumors and an increase in their circulation, correlating with poor prognosis in many instances. Consequently, the neutrophil/lymphocyte ratio emerges as a clinically relevant prognostic indicator in certain cancers [1]. Recent research has underscored the multifaceted role of neutrophils in cancer, influencing tumor growth, metastasis, cancer stem cell maintenance, exit from dormancy, cell cycle progression, immunosurveillance impairment, therapeutic resistance, antigen presentation, T cell response co-regulation, and antibody-dependent tumor cell clearance [4,5,6,7,8,9]. Neutrophils exhibit a remarkable plasticity, assuming both pro- and anti-tumor functions within the tumor microenvironment.

Recent reviews have comprehensively analyzed neutrophil functions in the context of cancer and tumor progression and resistance, shedding light on their clinical relevance for cancer patients [3,9,10]. In this review, we aim to consolidate the current understanding of the intricate interplay between neutrophils and cancer, with a focus on recent research elucidating their diverse functions at different stages of cancer metastasis.

## 2. Neutrophil Heterogeneity in Tumors

Tumor-associated neutrophils (TANs) play a pivotal role in the complex landscape of the tumor microenvironment (TME), exerting both stimulatory and suppressive effects on tumor development. The dual nature of their impact is contingent upon their activation status and the signals present in the TME. Originally categorized into low-density neutrophils (LDNs) and high-density neutrophils (HDNs) based on cell density, recent advancements in single-cell RNA sequencing (scRNA-seq) and mass cytometry by time-of-flight (CyTOF) have unveiled a more intricate and heterogeneous neutrophil subpopulation.

In liver cancer, scRNA-seq revealed 11 distinct neutrophil subsets, each displaying unique gene signatures regulated by transcription factors in a spatiotemporal manner. Notably, three subsets (Neu_01_MMP8, Neu_07_APOA2, and Neu_08_CD74) were predominantly enriched in tumors, signifying TANs. Three TAN subsets (Neu_01_MMP8, Neu_07_APOA2, and Neu_08_CD74) were associated with a worse prognosis, underscoring their pro-tumor functions. Integrating machine learning in cohorts of hepatocellular carcinoma (HCC) patients′ bulk-sequencing and single-cell sequencing data resulted in the development of a risk model termed the “neutrophil-derived signature” (NDS), which demonstrated superior accuracy in predicting prognosis compared to clinical variables [11].

In melanoma patients, mass cytometry identified seven neutrophil clusters, revealing terminally differentiated subsets that decreased during tumor progression and others that increased. Similarly, in lung cancer, mass cytometry identified subpopulations within LDN/HDN neutrophil subsets, with LDN subsets being highly enriched in the low-density fraction of advanced lung cancer patients. A unique CD66b^+^/CD10^low^/CXCR4^+^/PDL1 intermediate subset was exclusively found in advanced lung cancer patients, and its presence correlated with poorer prognosis [12].

Moreover, in non-small cell lung carcinoma (NSCLC), the activation of Smad3 in N2 TANs was negatively correlated with the N1 population and patient survival, suggesting heterogeneity in neutrophil tumor subset infiltration may impact tumor progression/regression [13]. Collectively, these findings underscore the critical importance of understanding neutrophil plasticity in the context of tumor progression, offering insights that may inform prognostic models and therapeutic interventions.

## 3. Pro-Tumoral Roles of Neutrophils

### 3.1. Neutrophil Recruitment in Tumors

The recruitment of neutrophils orchestrates a complex interplay influenced by various factors, including local signals at the injury/circulation site. As neutrophils migrate to the tumor site, alterations in their transcriptional profiles occur, shaped by the organs where they are recruited. These changes give rise to distinct phenotypic functions primarily driven by the varied expression of adhesion molecules and signaling through CXCL12-CXCR4 [14]. Notably, these studies underscore that neutrophils exhibit specialized functions in a tissue-specific manner.

Neutrophil recruitment is further intricately regulated by signaling between CXCR1 and CXCL8 at the recruitment site [15]. Once recruited, neutrophils exert an influence on T cell tumoricidal activity by enhancing regulatory T cell recruitment and activity within the tumor microenvironment (TME) across various cancer types [16,17]. Additionally, neutrophils secrete various factors, including neutrophil elastase (NE), matrix metalloproteinases (MMPs), reactive oxygen species (ROS), vascular endothelial growth factor (VEGF), and others. These factors can modify the TME, fostering conditions conducive to cancer cell growth and spread. Another mechanism by which neutrophil recruitment into the tumors can happen is by CXCL1/CXCR2 interaction, where the monomeric and dimeric CXCL1 can mediate neutrophil recruitment by binding with the glycosaminoglycans (GAC) and forming a gradient, helping the recruitment of neutrophils [18,19]. The GAC is a major component in the ECM and affects many properties of the tumor; one of the mechanisms can be the recruitment of the neutrophils via the CXCL1/CXCR2 interaction [20,21]. This can be more clearly seen in the study by Sengupta et al., where the tumor-conditioned media from highly aggressive breast cancer was able to recruit more neutrophils compared to a less aggressive one [22]. The authors showed that neutrophil recruitment and trafficking in breast cancer was mediated by CXCL1/2/3 in tandem with TGF-β. Moreover, the monomeric and dimeric forms of CXCL1 and CXCL2 and their ability to interact with CXCR2/GAG/Ackr1 may determine tissue-specific neutrophil recruitment [19]. Recently it was also shown that the CXCL1-CXCL2 heterodimer is potent in recruiting neutrophils [23].

Neutrophils induce a pro-tumoral environment by generating neutrophil extracellular traps (NETs), composed of modified chromatin adorned with bactericidal proteins. This NETosis, influenced by changes in mitochondrial metabolism, inhibits cytotoxic T lymphocyte access to cancer cells, promoting cancer progression. NETs play a crucial role in metastasis, with the dysregulation of gut microbiota being essential for colorectal cancer metastasis to the liver [24,25]. The interaction between NETs and CCDC25 on tumor cells enhances cancer cell motility via the activation of the IL-beta parvin pathway, facilitating metastasis [26]. Additionally, NETs activate dormant cancer cells through inflammation and are mechanistically linked to the proteolytic remodeling of laminin activated by integrin alpha3beta1 signaling [27].

Beyond its role in metastasis and angiogenesis, NETs impact immunotherapy by excluding cytotoxic CD8^+^ T cells from tumors, a process that can be reversed by blocking IL17 [28]. Colorectal cancer metastasis to the liver is primarily mediated by bacteria dissemination from the primary site, recruiting neutrophils via cytokines such as IL1-β, CCL2, TNFα, and IL6, ultimately forming a pre-metastatic niche [29]. Furthermore, CXCL1 and CXCL3 production, facilitated by TGF-β and SMAD3 signaling, leads to increased infiltration of immunosuppressive neutrophils in the liver, contributing to metastasis [30].

The immune response and outcomes are contingent on the type of neutrophils, i.e., immature recruited neutrophils or neutrophils aged at the site, resulting in differential ROS production and NET formation. Circadian rhythms, governed in part by neutrophil release from the bone marrow and the expression of transcription factors C/EBP and GATA1, influence this process [31,32]. CXCL12 signaling to CXCR4 further regulates neutrophil release, and recent studies suggest that CXCR2, a circadian rhythm-controlling factor, affects neutrophil granule loss and NET formation [33]. External factors like the microbiota impact neutrophil aging via TLR and MyD88 pathways, influencing its function toward a proinflammatory phenotype at the recruitment site [34].

Neutrophils recruited to the TME can be functionally classified into two types: N1, with anti-tumor functions induced by IFN-β and N2, with pro-tumor functions induced by TGF-β [35,36]. The N1 and N2 subsets exhibit plasticity during tumor progression, with the protumor effects of N1 associated with TNFα, ICAM-1, ROS, Fas, and reduced arginase expression. Conversely, the effects of N2 stem from MMP9, VEGF, arginase, and chemokines that promote tumor metastasis [37]. NK cells can modify neutrophil function via the IL-17A-neutrophil axis, dependent on IFN-γ. Interestingly, when neutrophils are depleted using the antimetabolite drug, the anti-tumor effect is highly dependent on the absence of NK cells, highlighting the crucial role of NK cells in neutrophil function [38].

### 3.2. Role in DNA Damage

In the context of lung cancer, the well-established association between smoking and increased lung cancer risk is mechanistically linked to the recruitment of neutrophils in the lungs, leading to the generation of elevated levels of reactive oxygen species (ROS) due to the presence of urethane in cigarette smoke. This heightened ROS production results in DNA damage, a pivotal factor in the progression toward cancer [39]. Notably, studies have demonstrated that G-CSF knock-out mice do not exhibit urethane-induced lung carcinogenesis, underscoring the specific role of neutrophils in this process [40].

Recent insights highlight that excessive ROS production and widespread transcription by neutrophils are the primary sources of NETosis, a process initiating chromatin decondensation. During NADPH-dependent ROS production, the key DNA repair protein PCNA translocates from the cytoplasm to the nucleus. Inhibiting this DNA repair protein formation impedes NETosis [41]. DNA, in this context, serves as an immunogenic mediator of inflammation alongside its role in NETosis. Toll-like receptor-9 (TLR9), cyclic GMP-AMP synthase (cGAS), NOD-like receptor protein 3 (NLRP3), and absence in melanoma 2 (AIM2) have been implicated in the formation of NETs [42].

Beyond the established role of ROS in NETosis, recent evidence highlights an ROS-independent mechanism of DNA damage by neutrophils, involving the release of microRNAs as microparticles. MicroRNAs such as miR-23a and miR-155 play a role in colorectal cancer (CRC) progression by inducing double-stranded breaks, inhibiting homology-directed repair carried out by Rad51 [43]. Targeting these microRNAs presents a promising avenue for inhibiting neoplasia. Interestingly, the same miR-155 can have both pro- and anti-tumor effects in different stages of CRC. In the early stages, miR-155 targets the Rad51 axis, decreasing homology-directed repair and reducing cancer cell survival. However, in later stages, miR-155 targets the non-homologous end-joining (NHEJ) pathway, increasing cancer cell survival by enhancing Ku70 foci formation [44,45].

Spontaneous NETosis is regulated by DNA polymerase Beta and DNA polymerase delta, controlling the baseline of ROS-induced DNA damage. ROS-induced NETosis leads to DNA lesions, halting genome-wide transcription and replication. This effect can be reversed by scavenging ROS or using NOX inhibitors. Inhibitors targeting later steps of DNA repair, predominantly involving PCNA and polymerase β/δ, increase NETosis levels, while inhibitors of earlier steps inhibit NETosis, suggesting that DNA damage in neutrophils has a regulatory role [46]. APE1, PARP, and DNA ligase are key proteins involved in this regulation. Targeting these DNA damage response pathway proteins using nuclear-penetrating anti-DNA autoantibodies significantly inhibits NETosis in both NADPH oxidase-dependent and -independent mice models [47].

DNA damage caused by neutrophils also regulates telomere dysfunction and senescence. The recruitment of neutrophils via the senescence-associated secretory phenotype results in an aged liver. Co-culturing neutrophils with fibroblast cells increases DNA damage and foci formation involving DNA damage response proteins γH2A.X (or 53BP1) and telomeres in fibroblasts [48].

### 3.3. Role in Tumor Cell Proliferation

Tumor initiation is a multi-step process involving oncogenic mutations in tissue progenitor cells, followed by proliferation and the eventual triggering of a pro-inflammatory response. Given the established role of neutrophils as key contributors to inflammatory responses, they play a crucial role in shaping tumor onset and progression, particularly within the tumor microenvironment (TME).

Following their development in the bone marrow, neutrophils undergo mobilization into the bloodstream and are subsequently recruited to tumor sites, facilitated by factors such as CXCL1. The interaction between CXCL1 and the abundance of CXCR2 on neutrophils leads to the accumulation of tumor-associated neutrophils (TANs) at these sites. This accumulation inhibits CD4^+^ and CD8^+^ T cells, primarily through the expression of myeloperoxidase (MPO) and Fas/FasL, thereby promoting tumor growth [49,50]. Recent findings highlight the potential prognostic significance of local CXCL1/2 expression within the TME [51]. The expression of CXCL1 is intricately regulated by miR-146a, influencing neutrophil recruitment and subsequently promoting tumor growth. Conversely, upregulating miR142 reduces tumor burden by diminishing neutrophil accumulation and enhancing CD8^+^ T cell presence in the tumor [52]. Tumor cells actively produce agonists of CXCR1 and CXCR2 to induce a neutrophil trap, preventing immune cytotoxicity and protecting tumors from CTL and NK cell attacks [53]. Increased neutrophil recruitment is often associated with an elevated expression of granulocyte colony-stimulating factor (G-CSF) and granulocyte-macrophage colony-stimulating factor (GM-CSF), which drive hematopoiesis toward granulocyte production [54].

Cancer cells, particularly cancer stem cells, produce chitinase-3-like protein 1 (CHI3L1), fostering neutrophil recruitment and exerting a pro-tumor function by modulating immunosuppressive T cell functions [55,56]. The release of IL-8 from dendritic cells is involved in the retention of neutrophils, forming a potential positive feedback loop between neutrophils and dendritic cells via CHI3L1 [56,57].

While both human and mouse neutrophils share consensus functions, some factors exhibit distinct effects. Notably, neutrophil elastase (ELANE) expression selectively targets cancer cells without harming normal cells [58,59]. The lungs, characterized by increased expression of platelet endothelial cell adhesion molecule-1 (PECAM-1), facilitate enhanced recruitment of neutrophils, allowing their trans-endothelial migration into the alveolar space and contributing to pro-tumorigenic effects [60].

### 3.4. Role in Extracellular Matrix (ECM) Remodeling

The Extracellular Matrix (ECM) constitutes a meticulously organized network of macromolecules, including proteins and polysaccharides, synthesized, and secreted by cells into the extracellular space. Major ECM components encompass collagen, elastin, fibronectin, laminin, aminoglycans, and proteoglycans. Neutrophils play a pivotal role in ECM remodeling through the release of specific matrix-remodeling enzymes such as neutrophil elastase and metalloproteinase, the formation of neutrophil extracellular traps (NETs), and the discharge of exosomes via secondary and tertiary particles [61].

The interstitial matrix and basement membrane of the ECM regulate tissue homeostasis and immune responses, influencing cell survival, growth, differentiation, and migration of immune cells in response to external stimuli [62]. Neutrophils expressing the integrin α6β1 receptor facilitate their transmigration across the ECM, primarily composed of collagen, laminin, and proteoglycans [63]. Neutrophil-derived proteases, including neutrophil elastase, matrix metalloproteinases, cathepsins, neutrophil gelatinase-associated lipocalin, NETs, and neutrophil-derived exosomes, modulate the ECM. The expression levels of neutrophil elastase can serve as a recurrence marker for differentiated thyroid cancer patients [59,64].

The activation of extracellular signal-regulated kinase (ERK) by neutrophil elastase contributes to the migration of colorectal cancer cells [65]. ECM remodeling, mediated by neutrophils, influences tumor cell survival and migration through Src/PI3K-dependent activation of Akt signaling, crucial for tumor cell dissemination in vivo [66]. Secreted neutrophil elastase sequentially cleaves laminin, activating dormant cancer cells by triggering integrin α3β1 signaling [27]. Stromal cell remodeling in the lung by neutrophil elastase promotes lung cancer [67].

Neutrophils, through heat shock–integrin signaling, participate in ECM healing in wounds. Increased expression of collagen-binding integrins αMβ2, ITGAM, and ITGβ2, coupled with neutrophil homing dependent on CXCR2, NOS, and LTB4R, enhances the healing process [68]. NETs released by neutrophils cleave fibronectin in lung alveolar tissues with the assistance of MMP9 [69]. In the mucosa of ulcerative colitis patients, neutrophil-derived NET elastase exhibits high elastinolytic activity, cleaving the therapeutic monoclonal antibody TNFα. Restoring balance is crucial, and the use of elafin is instrumental in achieving this [70]. In bone matrix scenarios, neutrophil-derived NET elastase disrupts the cartilage matrix, leading to the release of membrane-bound peptidylarginine deiminase-2 by fibroblast-like synoviocytes. Subsequent citrullination of cartilage fragments is internalized by fibroblast-like synoviocytes and presented to antigen-specific CD4^+^ T cells [69]. Neutrophil-derived MMP9 activates TGF-β, suppressing T cells [71].

NETs deposited by neutrophils facilitate neovascularization improve ischemia and hypoxia, activate CCDC25, and enhance metastasis through epithelial–mesenchymal transition (EMT) in gastric cancer. Blocking or knocking down CCDC25 and inhibiting NETs with DNaseI counteract these effects [72]. In diffuse large B-cell lymphoma (DLBCL), NETs recruited via the IL8-CXCR2 axis activate TLR9, increasing NF-κB and p38 pathways and promoting tumor proliferation and growth [73,74,75]. Neutrophil-derived APRIL is another factor contributing to increased tumor aggressiveness in DLBCL [76].

The ANGPT2 and Tie2 axis plays a role in vascular remodeling and tumor progression, with NETs increasing ANGPT2 expression in gastric cancer models [77]. NETs significantly stimulate endothelial cells, decrease CD31, ZO-1, and VE-cadherin expression, contract the cytoskeleton, increase intercellular area, and support proliferation and migration [72]. Neutrophil-derived MMP9 emerges as a major source of angiogenesis for cancer cells, being highly potent and uncomplexed with other MMP inhibitors [78].

### 3.5. Role in Immunosuppression

Due to their immunosuppressive characteristics, immature neutrophils within the tumor microenvironment (TME) are commonly identified as “polymorphonucler myeloid-derived suppressor cells (PMN-MDSC)” [79]. However, reports also indicate that mature neutrophils also exhibit an immunosuppressive phenotype [80,81]. In mice, these cells are defined as CD11b^+^Ly6G^+^Ly6C^low^, while in humans, they are identified as CD66b^+^CD14^−^CD11b^+^CD15^+^ [82]. Human PMN-MDSCs additionally express lectin-type oxidized LDL receptor-1 (LOX-1) [83]. PMN-MDSCs predominantly employ reactive oxygen species (ROS), peroxynitrite, arginase-1 (Arg1), and prostaglandin E_2_ (PGE2) to suppress the functions of T cells, B cells, and natural killer (NK) cells [84,85,86]. These MDSCs elevate arginase-1 levels, which degrade arginine, which is crucial for T cell activity via CD3z expression, modulating the interaction between CCR5 and its ligands [87].

Bianchi et al. highlighted cell-autonomous CXCL1 as a key mediator of spatial T cell restriction through interactions with CXCR2^+^ neutrophilic MDSCs in human pancreatic ductal adenocarcinoma (PDAC). Neutrophil-derived TNF regulates immunologic rewiring, CXCL1 overproduction from tumor cells, and T cell dysfunction [88]. Gong et al. revealed the tissue-specific immunosuppressive capacity of neutrophils, demonstrating that lung-infiltrating neutrophils mediated immunosuppression through CD140a^+^ mesenchymal cells (MCs) and prostaglandin-endoperoxide synthase 2 (PTGS2), the rate-limiting enzyme for PGE_2_ biosynthesis [89]. Furthermore, PMN-MDSCs undergoing ferroptosis exhibit heightened immunosuppression, producing oxygenated lipids and abundant PGE_2_, limiting the activity of human and mouse T cells [90].

Another avenue through which neutrophils acquire an immunosuppressive phenotype is the expression of PD-L1, interacting with PD-1 on T cells, inhibiting their proliferation and activation. In pancreatic ductal adenocarcinoma (PDAC), neutrophils deficient in purinergic receptor P2RX1 are mobilized and recruited at metastatic sites, accompanied by PD-L1 expression, contributing to immunosuppression [91]. Additionally, Arg1^+^ and PD-L1^+^ immunosuppressive neutrophils infiltrate the brain, facilitating brain metastasis development. Elevated expression of enhancer of zest homolog 2 (EZH2), phosphorylated at tyrosine-696 (pY696)-EZH2 by nuclear-localized Src tyrosine kinase, alters its binding preference, increasing c-JUN expression and promoting the recruitment of immunosuppressive neutrophils into the brain [92]. In gastric cancer, cancer cell-derived extracellular vesicles (GC-EVs) transport high-mobility group box-1 (HMGB1), inducing PD-L1 expression on neutrophils and inhibiting T cell immunity [93]. In gastric cancer, activated neutrophils exhibit increased programmed death-ligand 1 (PD-L1) expression, induced by cancer cell-derived GM-CSF. This upregulation of PD-L1 leads to the suppression of normal T cell immunity [94]. The combination of interleukin-17 (IL-17) and G-CSF contributes to heightened hemostasis and neutrophil inflammation [95]. Subtypes of IL-17, such as IL-17C from epithelial cells, further promote neutrophil recruitment in lung tumor growth, influenced by the lung microbiota [96].

### 3.6. Role in Cytokines/Chemokines Production

Neutrophils constitute a diverse reservoir of cytokines, chemokines, and growth factors that exert significant influence on the tumor microenvironment, consequently shaping tumor outcomes.

In a study by Queen et al., a co-culture of neutrophils with human breast cancer cell lines led to the release of oncostatin M (OSM) by neutrophils, fostering angiogenesis through the induction of vascular endothelial growth factor (VEGF) [97]. In the context of intrahepatic cholangiocarcinoma (ICC), tumor-associated neutrophils (TANs) exhibited a pro-tumorigenic character by producing elevated levels of oncostatin M, activating STAT3 signaling in ICC cells [17]. TGF-β released by neutrophils in breast cancer has been implicated in promoting tumor cell resistance to gemcitabine by inducing epithelial-to-mesenchymal changes in tumor cells [98]. Furthermore, breast cancer cell–neutrophil interaction regulated the pro-tumorigenic phenotype of neutrophils, leading to cancer progression and therapy resistance. Neutrophils exposed to breast cancer cell-derived supernatant displayed heightened levels of interleukin-1β (IL-1β), CC-chemokine ligand-2-4 (CCL2, CCL3, and CCL4), inducible nitric oxide synthase (iNOS), and matrix metallopeptidase-9 (MMP9) [99].

In gastric cancer, TANs were widely distributed in gastric tissues and correlated with tumor stage and poor prognosis. Notably, these TANs produced IL-17A, enhancing the migration, invasion, and epithelial–mesenchymal transition (EMT) of gastric cancer cells through the activation of the Janus kinase 2/signal transducers and activators of transcription (JAK2/STAT3) pathway [94]. Within a lung cancer model, local microbiota activated neutrophil release of IL-1β and IL-23, inducing lung-resident γδ T cells that promoted inflammation and tumor growth [100]. Neutrophils played a role in facilitating the extravasation of tumor cells in lung parenchyma through the secretion of IL-1β and matrix metalloproteinases [101]. Moreover, TANs, as demonstrated by Zhou et al., produced bone morphogenetic protein-2 (BMP2) and transforming growth factor beta2 (TGF-β2), inducing stem cell characteristics in hepatocellular carcinoma (HCC) cells. These stem-like HCC cells, in turn, recruited more TANs via hyperactive NF-κB signaling, establishing a positive feedback loop that increased the proliferation rate of HCCs [102].

### 3.7. Role in Neutrophil Extracellular Trap Formation

The exploration of neutrophil extracellular traps (NETs) has significantly advanced since their initial observation in 1996 [103] and the subsequent discovery by Brickman et al. in 2004 [104]. These intricate web-like structures, composed of DNA strands and proteins, serve as novel extracellular killing mechanisms adapted by neutrophils to trap and eliminate microorganisms [105,106]. Over almost two decades, ongoing research has unveiled a deeper understanding of NETs’ potential mechanisms and implications in both physiological processes and various pathophysiologies. The involvement of NETs in cancer progression was initially demonstrated when induced by sepsis in the context of liver and lung metastasis [107]. A recent genome-wide association study (GWAS) and exome sequencing linking genetic variants of patients with chronic diseases to circulating NET levels found an association between genetic variants in 10 genes and NET levels, with cancer emerging as the top disease linked to NET-associated genes [108]. NETs’ association has been documented across various cancer types, including breast cancer [109], pancreatic cancer [110], lung cancer [111], ovarian cancer [112], bladder cancer [113], gastric cancer [73], skin cancer [114], and colorectal cancer [115].

Nevertheless, studies in both animal models and patient blood and tumors suggest that NETs may function as either pro- or anti-tumoral factors, contingent on the immune system status or tumor microenvironment [116,117]. Indications of NETs being protumorigenic point to their involvement in cancer progression, metastatic spread, and cancer-associated thrombosis [118,119]. Several mechanisms underline the pro-tumor effects of NETs. The main NET components, neutrophil elastase (NE) and cathepsin G (CG), activate dormant cancer cells by degrading the extracellular matrix (ECM) protein laminin [120]. This process generates an epitope binding to tumor integrins, promoting cancer cell extravasation, proliferation, and metastasis [27,121,122]. NETs enhance angiogenesis, thereby mediating cancer proliferation [123], entrapping cancer cells, acting as adhesion substrates to facilitate metastatic dissemination [107,124], promoting endothelial-to-mesenchymal transition [125], and shielding tumor cells from CD8^+^ T cells and natural killer (NK) cell-mediated cytotoxicity [53].

Contrastingly, studies have shown that NETs can exert anti-tumor effects. NET components such as myeloperoxidases (MPO), proteinases, and histones demonstrate the ability to eliminate tumors, inhibit tumor growth, and hinder metastasis [126,127]. Intriguingly, there exists a positive feedback loop between tumor-induced NETosis and NET-driven tumor growth, with various components in the tumor microenvironment acting as NET inducers. Cytokines like G-CSF [128], IL-1β [129], Interleukin-8 [73], and CXCR1 and CXCR2 agonists [53] have been demonstrated to stimulate cancer-associated NETosis. In orthotopic tumors, the induction of NET formation via IL17, through the induction of CXCR1/2 agonist chemokines, was observed. This attracted neutrophils, leading to NETosis inhibition, increased infiltration of activated CD8^+^ T cells, and heightened sensitivity to PD-1 blocking mAbs [28]. Another study by Wang et al. revealed that the pseudo serine protease PRSS35 induces CXCL2 degradation, attenuating neutrophil recruitment to tumors and NET formation, ultimately suppressing hepatocellular carcinoma (HCC) progression [130]. Tumor-induced HMGB1 has also been identified as a stimulator of cancer-associated NETosis [131]. The HMGB1/RAGE/IL-8 axis plays a crucial role in neutrophil recruitment into the tumor microenvironment (TME) and NETosis as HMGB1 was found to bind the RAGE receptor on glioma cells, activating NF-κB and upregulating IL-8 expression—an influential neutrophil chemoattractant [132]. Additionally, tumor cells have been shown to upregulate extracellular vesicle (EV) production, communicating with the stroma to create a tumor-permissive environment. Specifically, human melanoma cell-derived EVs promote neutrophil recruitment and NET production by signaling through the CXCR2/PI3K-AKT axis [114]. Recent findings by Li et al. demonstrated that fibroblast growth factor 19 (FGF19)-induced inflammatory cancer-associated fibroblasts (iCAFs) promote neutrophil infiltration and mediate NET formation in liver metastatic niches via the production of complement C5a and IL-1β, accelerating the liver colonization of colorectal cancer (CRC) cells [133]. Furthermore, Yao et al. reported that NETs interact with TLR9 to decrease merlin phosphorylation, contributing to triple-negative breast cancer (TNBC) cell ferroptosis resistance and promoting TNBC progression [134].

### 3.8. Role in ROS Generation

Neutrophil-derived reactive oxygen species (ROS) play a pivotal role in fostering mutational burden, thereby propelling cancer initiation and advancement. This occurs through the promotion of oxidative DNA damage in critical tissues such as the lungs and intestines [135]. In a lung tumor model, researchers identified the impact of neutrophil NOX2-derived ROS in facilitating tumor colonization through an IL-1β-dependent pathway [136]. Another study demonstrated that neutrophil-produced ROS induced DNA damage in the lungs, thereby fostering tumor formation, especially under co-treatment with carcinogens [40].

Neutrophils can inhibit the activity of T cells within the TME. The reduced levels of glucose within the TME force the neutrophils to use mitochondrial-derived NADHP for ROS production, which then inhibits the CD4^+^ T cells and has been shown using a 4T1 tumor-bearing mice model. This mainly occurs by decreasing the viability of these T cells and decreasing the T cells’ ability to produce IFN-γ [137]. Moreover, the ROS derived from the neutrophils inhibit IL17-producing gamma delta T cells, which can act as a major pro-tumoral agent. This mainly occurs as a virtue of reduced production of antioxidant glutathione in these gamma delta T cells, rendering them susceptible to this neutrophil-derived ROS [138]. In the case of circulating neutrophils, they can still inhibit the activity of the T cells by suppressing their proliferation. This inhibition in the immunological synapse requires the expression of integrin Mac-1 on the neutrophil’s surface [139]. However, the immature neutrophils have less T cell suppression activity compared to the mature subset in the case of cancer and the increase in circulating immature neutrophils in the bloodstream of cancer patients represents a mere correlation with the suppressive activity [140]. This has also been shown by Minns et al., where, if the T cell interacts with the neutrophils during the early stage of activation, they are suppressive, but if the T cell has previously been activated, they are not susceptible to the neutrophil-mediated suppression [141].

In the context of breast cancer, tumor-associated neutrophils strategically leverage ROS production to subdue T cells by engaging in oxidative mitochondrial metabolism. This priming of neutrophils, induced by the tumor, allows these cells to sustain an immunosuppressive phenotype, particularly within a glucose-deprived tumor microenvironment [137]. Neutrophil-derived ROS can also exert immunosuppressive effects on T cells by diminishing CD3ζ chain and cytokine expression [142]. Furthermore, neutrophil-derived hydrogen peroxide (H_2_O_2_) exhibits the capability to inhibit natural killer (NK) cell function, diminishing tumor clearance and promoting lung colonization in a mouse model of breast cancer metastasis [143]. In mice with a myeloid cell-specific deletion of glutathione peroxidase 4 (Gpx4), a crucial ROS scavenger, more invasive colon tumors developed after repeated injections of the carcinogenic agent azoxymethane. This outcome resulted from excessive ROS production by Gpx4-deficient myeloid cells, amplifying the mutational load of colonic epithelial cells and consequently rendering arising tumors more aggressive [135].

In a subcutaneous cancer mouse model, the expression of inducible nitric oxide synthase (iNOS) by infiltrating neutrophils closely correlates to the number of hypoxanthine phosphoribosyl transferase (Hprt) mutations. These mutations significantly contribute to the genetic abnormalities associated with tumor progression [144]. In the context of inflammatory bowel disease, activated neutrophils release microparticles containing pro-inflammatory microRNAs, including miR-23a and miR-155, resulting in DNA double-strand breaks and subsequent genomic instability. Remarkably, this process occurs in an ROS-independent manner. Evidence of such metabolic shifts is increasingly apparent across various cancer contexts and is intricately linked to immunosuppressive capabilities [43].

## 4. Anti-Tumoral Roles of Neutrophils

### 4.1. Role in Tumor Cell Killing

Neutrophils, like other immune cells, exhibit diverse and sometimes opposing functions throughout various stages of cancer. They employ surface receptors, such as Fc receptors, to recognize antibody-opsonized tumor cells. Notably, cancer patients often undergo treatment with tumor-targeting monoclonal antibodies (mAbs). The interaction of Fc receptors promotes neutrophil activation and effector functions. Therefore, opsonizing tumor cells with tumor-associated antigens (TAA)-targeting antibodies enables neutrophils to identify and eradicate tumor cells. Neutrophils play a crucial role in cancer cell elimination through antibody-dependent cellular cytotoxicity (ADCC) and antibody-dependent cellular phagocytosis (ADCP) [145]. Additionally, IgA antibodies, in particular, demonstrate superior cancer cell-killing capabilities compared to IgG. This heightened efficacy is linked to the checkpoint inhibition of the CD47–SIRPα interaction [146,147,148]. SIRPα acts as a signal for inhibiting ADCC by cancer cells, presenting it as a potential target for enhancing neutrophil-mediated ADCC.

Another mechanism facilitating the interaction between neutrophils and various types of tumor cells is Mac-1-dependent cellular contacts, crucial for neutrophil-mediated tumor cell killing [147,149]. In vitro studies revealed close interactions between neutrophils and trastuzumab (IgG)-opsonized SK-BR-3 cells, leading to tumor cell killing [147]. Another study highlighting neutrophil-killing potential demonstrated that combination therapy involving tumor necrosis factor, CD40 agonist, and a tumor-binding antibody induced rapid mobilization and tumor infiltration of neutrophils [150]. The activation of complement component C5a further stimulated neutrophils to produce leukotriene B4, promoting reactive oxygen species production via xanthine oxidase. This cascade resulted in oxidative damage and T cell-independent clearance of multiple tumor types [151].

Neutrophils employ various mechanisms to induce the death of tumor cells, including apoptosis, necrosis, and autophagy. One such pathway through which neutrophils may trigger tumor cell death is the Fas–Fas ligand (FasL) pathway. The Fas receptor, a death receptor (DR) expressed on various cells, undergoes trimerization of the death domain upon cross-linking with its ligand FasL. This activation leads to the subsequent initiation of the caspase pathway and induction of apoptosis [152]. Neutrophils express FasL, a member of the TNF superfamily, and a study suggests that they may release soluble FasL, thereby inducing tumor cell death via the Fas/FasL pathway [153].

TNF-related apoptosis-inducing ligand (TRAIL), another member of the TNF superfamily, interacts with various TRAIL receptors, including death receptors DR4 (TRAIL-R1) and DR5 (TRAIL-R2), as well as decoy receptors DcR1 (TRAIL-R3) and DcR2 (TRAIL-R4) [145]. IFN-stimulated neutrophils are reported to express TRAIL, leading to the induction of tumor cell apoptosis [154]. In another study, evidence of neutrophil-mediated tumor cytotoxicity was observed in IgG-opsonized tumor cells, resulting in necrosis-like cell death and the release of cellular contents. The incubation of human mammary carcinoma cells with human neutrophils and anti-FcαRIxHer2/neu BsAbs revealed autophagic structures and LC3B+ autophagosomes in different human epithelial carcinoma cells, ultimately resulting in neutrophil-mediated autophagic tumor cell death [155].

### 4.2. Role in Trogocytosis and Trogoptosis of Tumor Cells

Trogocytosis, an intricate mechanism utilized by immune cells, involves the acquisition of membrane proteins from other cells through an endocytic process. During trogocytosis, the immunological synapse facilitates the transfer of plasma membrane fragments from one cell to another [156]. Neutrophils, engaging in close contact with rituximab-opsonized CLL B cells, absorb tumor cell membrane fragments, resulting in a notable decrease in CD20 expression in the tumor cells [157]. Additionally, when neutrophils are incubated with epratuzumab (anti-CD22)-opsonized Daudi B lymphoblast cells, there is a transfer of CD22 from tumor cells to neutrophils [158]. Other tumor-derived membrane proteins, including CD19 and CD79b, have been identified on neutrophils following co-incubation with tumor cells. Likewise, after incubation with rituximab-opsonized B cells, neutrophils express both CD19 and CD20 [159]. Given the expression of various Fc receptors (FcRs) on neutrophils, they are capable of trogocytosis of opsonized tumor cells, forming close cell–cell interactions and a Mac-1-dependent cytotoxic synapse. Subsequently, neutrophils engage in trogocytosis, nibbling small fragments of the cell membrane and cytosol from the tumor cell. Horner et al. proposed that neutrophil trogocytosis may lead to opsonized Raji tumor cell destruction [160]. Matlung et al. demonstrated that neutrophils employ antibody-mediated trogocytosis as a mode of cancer cell destruction, both in vitro and in vivo. This process involves CD11b/CD18-dependent conjugate formation, resulting in trogoptotic (lytic) death of antibody-opsonized solid cancer cells. Interestingly, inhibiting neutrophil trogocytosis prevents trogoptosis of target cells, while enhancing trogocytosis through interference with CD47–SIRPα interactions enhances killing. This highlights a potential avenue for optimizing the clinical efficacy of antibody therapy in cancer [147].

### 4.3. Role in Tumor Cell Cytotoxicity via Neutrophil Degranulation

Neutrophils, often referred to as granulocytes due to their densely packed cytoplasm filled with numerous cytotoxic granules, house an array of substances, including myeloperoxidase (MPO), neutrophil elastase (NE), lactoferrins, gelatinase, and defensins. Neutrophil degranulation is a process by which neutrophils release their granules, including cytotoxic antimicrobial proteases as a way of non-oxidative killing mechanism. Degranulation can occur at the plasma membrane for extracellular release (killing extracellular microorganisms) or in the phagosome for intracellular delivery (killing intracellular microorganisms) [161]. Neutrophil granules can be broadly classified into four main types: secretory, tertiary, secondary, and primary. Secretory granules include plasma proteins and Fc and complement receptors. Tertiary granules include matrix metalloproteases, such as matrix metallopeptidase 9 (MMP9). Secondary granules involve lysozyme, pre-cathelicidin, and lactoferrin. Primary granules include myeloperoxidase, defensins, elastase, and azurocidin [161]. Although these granules are mostly known for their pro-inflammatory and antimicrobial functions, some studies have suggested that these granules also possess the capability to induce tumor cell cytotoxicity [162,163]. A recent study demonstrated that human neutrophils release catalytically active neutrophil elastase (ELANE), which proteolytically cleaves the CD95 death domain, binding it to histone H1 in cancer cells [27]. The released ELANE from neutrophils exhibits an abscopal effect, mediated by CD8^+^ T cells [58].

### 4.4. Role in Crosstalk with Adaptive Immune System

During the initial stages of lung cancer, neutrophils assume a stimulatory role, bolstering T cell function, leading to heightened T cell proliferation and an increased release of IFN-γ. This sequential cascade results in elevated proinflammatory factors and upregulation of costimulatory molecules on T cells [164]. Studies by Fridlender et al. showcased that blocking TGF-β facilitates neutrophil recruitment, thereby exhibiting anti-tumor activity by supporting cytotoxic T cell-mediated responses [36,165]. Similar antitumor effects of neutrophils, achieved through TGF-β inhibition, were observed in colorectal cancer (CRC) [166].

The anti-tumor activity of neutrophils can be further enhanced by obstructing the PD-L1/PD-1 axis on cancer cells [167]. Neutrophil recruitment, crucial for nitric oxide (NO) production, contributes to the effective killing of cancer cells, particularly evident in lung cancer. This process is mediated by the MET and HGF receptors, where blocking MET enhances neutrophil recruitment while concurrently suppressing T cell functions and expansion [168,169,170].

Neutrophils also exert their anti-tumor effects by modulating tumor-associated microbiota through the regulation of IL17, thereby promoting intratumor B cells [171]. However, conflicting results in studies using the same transplanted cell lines indicate context-dependent functions of neutrophils. Discrepancies in experimental methods, sampling time, antibodies used for neutrophil depletion, and cancer progression stages may contribute to these variations, underscoring the necessity for further investigation. In a recent study by Yam et al., tumor neutrophils were found to undergo a transformation from a wound-healing to an acutely activated cytotoxic phenotype. Microbial-activated neutrophils upregulated chemokines, facilitating CD8^+^ T cell recruitment. They transitioned from sessile VEGF producers to highly motile neutrophils, forming neutrophil-rich domains in tumors, thereby repressing tumor growth [172].

### 4.5. Role as of Antigen-Presenting Cells

Tumor-infiltrating neutrophils observed in different cancers help in transporting the tumor antigens from the tumor site to the draining lymph nodes, where they are presented to the T cells to initiate the immune response. Along with the expression of CD40 and CD80, this phenomenon of neutrophils is attributed to its potent phagocytosis characteristic [173,174,175]. They are the earliest immune cells to be presenting the tumor antigen. Neutrophil antigen presentation characteristic is conferred by GM-CSF, IFN-γ, and IL-3 [176,177,178]. However, higher expression of the MHC molecule for the antigen presentation on the neutrophils requires cues from the activated T cells, and innate signals like TLR or DAMPs are not sufficient for this [74,179,180]. The neutrophil’s increased ability to process antigen and present it is important for priming Th1 and Th17 cells in an antigen-specific manner [181]. The neutrophil’s APC role primarily occurs as a virtue of decreased DC and T cell migration into the tumor site due to the increased neutrophil-secreted thromboxane A2 [182,183]. Although nAPC presents the antigens, it lacks the conventional ability of robust signaling to the T cells as seen for professional DC. However, this ability to strongly activate T cells at par with the DC can be increased by endocytosis of the antibody-antigen complex via FcγR in vivo and has been shown to elicit CD8^+^ T cell-dependent anti-tumor immunity [184]. The role of transcription factor PU.1 is critical for this enhanced ability of neutrophils to work as an APC. This approach is gathering much-needed attention as an immunotherapeutic strategy to overcome the drawbacks associated with the use of conventional DC use. A novel approach that enhances immune-mediated tumor regression by activating the noncanonical antigen presentation by the neutrophils makes use of radiotherapy–radiodynamic therapy (RT-RDT) with nanoscale metal–organic frameworks (nMOFs). This has also been found to increase the surface expression of CD80 and CD86 as well as MHCII molecules on the neutrophils for an effective cross-presentation of antigens [185].

## 5. Clinical Relevance and Future Directions

**Figure 1:**
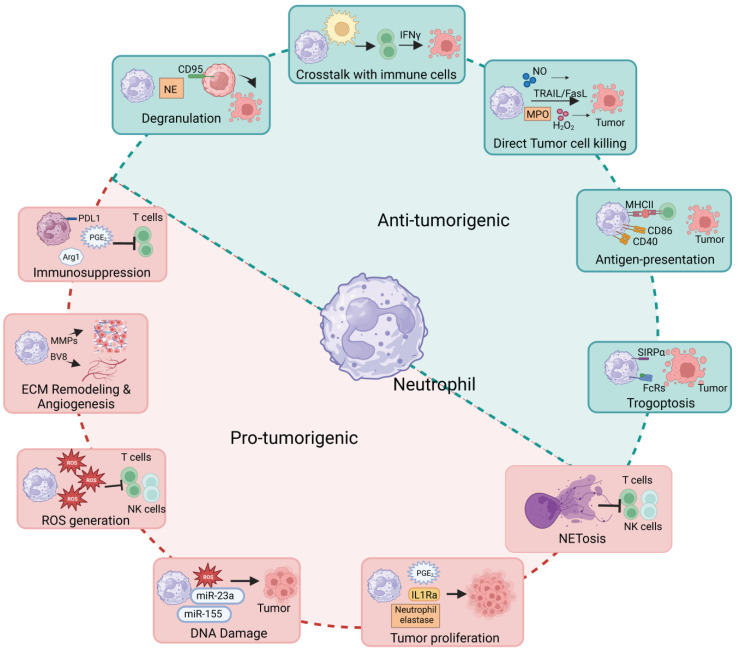
The multifaceted role of neutrophils in the tumor microenvironment: Neutrophils can act as both pro-tumorigenic and anti-tumorigenic, employing various functions depending on the disease stage and their location (as described in detail in the text). ROS-Reactive Oxygen Species; DNA-Deoxyribonucleic acid; IL1Ra-Interleukine-1 Receptor Alpha; NK cells-Natural Killer Cells; SIRPa- Signal Regulatory Protein alpha; MHCII- Major Histocompatibility complex II; PGE1-Prostaglandin E1; TRAIL/FasL-Tumor Necrosis Factor-Related Apoptosis Inducing Ligand/Fas Ligand; NO-Nitric Oxide; Arg- Arginase; PDL1-Programmed death-ligand 1; MMP- Matrix metalloproteinases; MPO-Myeloperoxidase; FcRs- Fc receptors. Created with BioRender.com (accessed on 25 January 2024).

Neutrophils, with their dynamic responsiveness to infection, inflammation, and shifts in homeostasis, represent complex and intriguing cells. Recent research has unveiled their dual impact on the tumor microenvironment (Figure 1), yielding both favorable and adverse outcomes contingent on tumor types and disease stages. However, the challenges stem from their transient lifespan, low RNA content, and adaptable nature, presenting a lack of distinct markers for discerning between different neutrophil subpopulations [186]. Despite substantial strides in comprehending neutrophil biology, functions, and heterogeneity, numerous questions persist regarding their roles in cancer.

Of note, currently, there are several undergoing preclinical and clinical studies to exploit neutrophil survival and recruitment and their potential anti-tumor functions as a therapeutic strategy in various cancers.

In a phase I study of patients with stage III head and neck squamous cell carcinoma, DS-8273a (an agonistic death receptor 5 (TRAIL-R2) antibody) was used to selectively target MDSCs. Treatment with DS8273a reduced the number of circulating PMN-MDSCs (CD11b^+^/CD14^+^/CD33^+^/CD15^+^; low-density fraction cells) in patients [187]. Moreover, in a phase II clinical trial, gemcitabine increased the efficacy of nivolumab by killing MDSCs to reduce immunosuppression in stage IIIB NSCLC (NCT03302247). In a phase II clinical trial of patients with stage IV CRC, 5-FU plus bevacizumab and anakinra resulted in an improvement of median progression-free survival [188]. However, these drugs also depleted other subsets of neutrophils as side effects causing neutropenia.

Moreover, current research indicates their involvement in cancer therapy as drugs targeting oncogenic signaling in tumor cells through c-MET, a receptor tyrosine kinase, not only impedes tumor progression but also blocks neutrophil recruitment into tumors and the draining lymph nodes. This absence of neutrophils facilitates T cell expansion, fostering tumor elimination [189]. Similarly, therapies targeting the CXCR2 signaling pathway, which plays a critical role in the trafficking of neutrophils from the bone marrow into the bloodstream and subsequently into the peripheral tissues, are being actively investigated in various cancers. AZD5069, a CXCR2 inhibitor that is used to reduce absolute neutrophil recruitment in bronchiectasis patients [174], is also under a phase I/II study in combination with durvalumab in patients with advanced hepatocellular carcinoma (HCC) [190]. Similarly, clinical trials targeting SX-682, a small-molecule inhibitor of CXCR1 and CXCR2 in combination with pembrolizumab (anti-PD-1 immunotherapy), are undergoing for metastatic or recurrent stage IIIC or IV non-small cell lung cancer [NCT05570825], metastatic melanoma [NCT03161431], and metastatic colorectal cancer (mCRC) [NCT06149481]. Another mechanism that exploits the tumor-killing ability of neutrophils is using TGF-β-blocking antibodies either as monotherapy or as combination therapy with [191] fresolimumab, which has been shown to be used in patients with melanoma [192], renal cell carcinoma [193], and metastatic breast cancer [194]. Moreover, fresolimumab and stereotactic ablative radiotherapy is currently being used in early-stage non-small cell lung cancer in phase I/II trials [NCT02581787]. Similarly, galunisertib is a small-molecule selective inhibitor of TGFβ receptor I that has been used in HCC [195]. In antibody cancer therapy, neutrophils engage with cancer cells, initiating antibody-dependent cellular cytotoxicity (ADCC) and eliminating cancer cells through trogocytosis or potentially degranulation [147].

While existing research predominantly emphasizes targeting the immunosuppressive or other pro-tumoral functions of neutrophils, there is a burgeoning yet promising avenue for exploring their cancer-killing capabilities. A nuanced approach is vital, considering the integral role neutrophils play in innate immunity. Utter suppression of this cell population could elevate infection risks. Hence, identifying markers to selectively target protumor neutrophil subtypes or intervening in signaling pathways that drive their protumorigenic nature emerges as a more prudent and promising therapeutic strategy against cancer. Future studies aimed at unraveling these intricate mechanisms will undoubtedly redefine neutrophils’ roles in cancer growth and metastasis, paving the way for innovative treatments that modulate their behavior in a context-dependent manner.
